# Effects of Age and Size on Xylem Phenology in Two Conifers of Northwestern China

**DOI:** 10.3389/fpls.2017.02264

**Published:** 2018-01-12

**Authors:** Qiao Zeng, Sergio Rossi, Bao Yang

**Affiliations:** ^1^Key Laboratory of Desert and Desertification, Northwest Institute of Eco-Environment and Resources, Chinese Academy of Sciences, Lanzhou, China; ^2^University of Chinese Academy of Sciences, Beijing, China; ^3^Département des Sciences Fondamentales, Université du Québec à Chicoutimi, Chicoutimi, QC, Canada; ^4^Key Laboratory of Vegetation Restoration and Management of Degraded Ecosystems, Guangdong Provincial Key Laboratory of Applied Botany, South China Botanical Garden, Chinese Academy of Sciences, Guangzhou, China

**Keywords:** cambium activity, cell differentiation, *Juniperus przewalskii*, *Pinus tabulaeformis*, radial growth, wood anatomy, xylogenesis

## Abstract

The climatic signals that directly affect the trees can be registered by xylem during its growth. If the timings and duration of xylem formation change, xylogenesis can occur under different environmental conditions and subsequently be subject to different climatic signals. An experimental design was applied in the field to disentangle the effects of age and size on xylem phenology, and it challenges the hypothesis that the timings and dynamics of xylem growth are size-dependent. Intra-annual dynamics of xylem formation were monitored weekly during the growing seasons 2013 and 2014 in Chinese pine (*Pinus tabulaeformis*) and Qilian juniper (*Juniperus przewalskii*) with different sizes and ages in a semi-arid region of northwestern China. Cell differentiation started 3 weeks earlier in 2013 and terminated 1 week later in 2014 in small-young pines than in big-old pines. However, differences in the timings of growth reactivation disappeared when comparing the junipers with different sizes but similar age. Overall, 77 days were required for xylem differentiation to take place, but timings were shorter for older trees, which also exhibited smaller cell production. Results from this study suggest that tree age does play an important role in timings and duration of growth. The effect of age should also be considered to perform reliable responses of trees to climate.

## Introduction

The climatic and environmental signals that directly affect trees can be registered by tree rings during their growth period (Frankenstein et al., 2005). Based on this principle, tree-ring records are both important and useful in reconstructing past climates as well as predicting the dynamics of forest ecosystems under future climate conditions (Rathgeber et al., 2000; Carrer and Urbinati, 2004; Yang et al., 2014; Wang et al., 2017). However, especially in the archeological wood material, the majority of tree-ring proxy data used in the climatic reconstruction consists of chronologies from trees with different ages and sizes (Büntgen et al., 2012). As tree-ring width generally increases during the youth stage and gradually deceases as trees grow older and larger (Autin et al., 2015), the information of growth-climate relationship extracted from the long-term variations of tree-ring series may be affected by this age-related growth trend (Briffa and Melvin, 2011). This raises the question of whether and how age and size factors affect the relationship between growth and climate.

Cambium maintains the ability to produce new cells throughout the tree's lifespan. Changes in xylem phenology occur as a tree ages and subsequently increases in size (Rossi et al., 2008; Li et al., 2013). At the alpine timberline, xylem phenology was 2–3 weeks longer for adult trees (50–80 yr) compared to old trees (200–350 yr), resulting in greater cell production (15–20%) along the radius of adult trees (Rossi et al., 2008). However, it remains unclear whether variation in xylogenesis during tree growth is associated with tree age or size, and this is because older trees are also larger in size (Rossi et al., 2008). Thus, there is an urgent need to disentangle the effects of age and size on wood formation to better understand the drivers of tree growth.

Compared to mature trees, the unfolding of spring leaves started 10–40 d earlier in seedlings (Vitasse, 2013). Vieira et al. (2013) investigated trees of the same age and size under identical localized conditions. Their results demonstrated that the timings of xylem phenology changed with cell production, but not with age or size *per se*. Rathgeber et al. (2011) found that trees of different social status and sizes, growing in even-aged stands, exhibited different xylem phenology. Compared to intermediate and suppressed trees, the cambial reactivation of dominant trees exhibited an earlier onset and a later termination and, therefore, a longer duration of xylem growth. De Luis et al. (2009) investigated an even-aged conifer plantation (*Pinus halepenisi* and *Pinus pinea*) under semi-arid Mediterranean climate conditions. The faster-growing pines were able to resume cambial activity in response to early autumn precipitation (De Luis et al., 2007); thus, they speculated that both size and growth rate affected their sensitivity to rain events. Some studies applied grafting techniques to separate the effect of size from the effect of age (Mencuccini et al., 2005, 2007; Abdul-Hamid and Mencuccini, 2009). According to this method, it has been suggested that photosynthesis and tree growth decline with increasing tree size (Abdul-Hamid and Mencuccini, 2009). Similarly, Mencuccini et al. (2005) concluded that tree size, not age, mediated changes in growth, carbon assimilation, and leaf biochemistry. Based on available literature, it seems that changes in phenology and the growth-climate relationship may mostly be associated with the size rather than the age of trees.

The main objective of this study was to quantify the effects of age and size on xylem phenology. Based on this context, we monitored the intra-annual dynamics of wood formation in conifer species during the 2013 and 2014 growing seasons in northwestern China. Trees were selected based on their ages and sizes to disentangle the effects of these two factors on xylem formation. We investigated cambial phenology at a weekly resolution to challenge the hypothesis that timings and dynamics of xylem growth are size-dependent.

## Materials and methods

### Study sites

**Figure 1:**
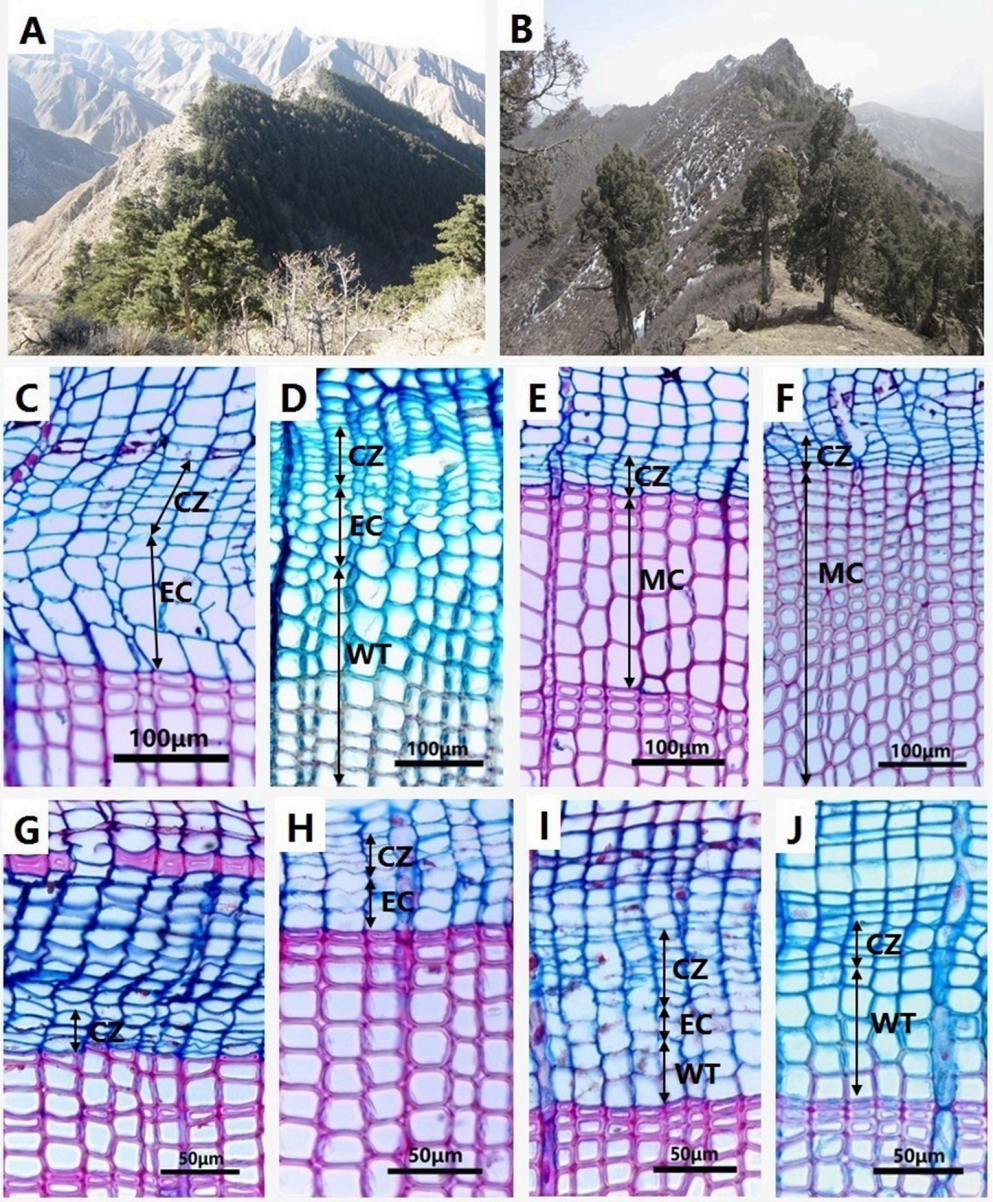
Chinese pine (*Pinus tabulaeformis*) forest in HSM **(A)** and Qilian juniper (*Juniperus przewalskii*) forest in SDL **(B)**. **(C)** Active cambium (CZ) and enlarging cells (EC) of big-old pines on 13 June. **(D)** Enlarging (EC) and wall-thickening (WT) cells of small-young pines on 12 July. **(E)** Mature cells (MC) of big-old pines on 29 August. **(F)** Mature cells (MC) of small-young pines on 2 September. **(G)** Dormant cambium (CZ) of big-old junipers on 12 May. **(H)** Enlarging cells (EC) of small-old junipers on 8 June. **(I)** Enlarging (EC) and wall-thickening cells (WT) of big-old junipers on 9 July. **(J)** Wall-thickening cells (WT) of small-old junipers on 21 July.

Two sites were selected in the semi-arid region of northwestern China. The first site (named HSM) was located on the Hasi Mountains in Jingyuan Country (Gansu Province) (37°02′ N, 104°28′ E, 2,456 m a.s.l.). Chinese pine (*Pinus tabulaeformis* Carr.) is the dominant tree species in this region (Figure 1A), which grows on northern slopes at altitudes between 2,200 and 2,700 m a.s.l., associated with *Picea crassifolia* (10–20%; Li, 2003). The forest canopy closure is 0.4–0.7 and soils are characterized as gray-cinnamon soil (Li, 2003). The second site (named SDL) was located at the Sidalong Forestry Station, situated on south-facing slopes in the central Qilian Mountains in the upper reaches of the Heihe River (38°26.64′ N, 99°56.03′ E, 3,550 m a.s.l.). We established this second study plot at the upper distribution limit of Qilian juniper (*Juniperus przewalskii* Kom.), with a forest canopy closure of 0.5 (Figure 1B; Wang et al., 2015). Shrubs such as *Caragana jubata, Dasiphora fruticosa*, and *Nitraria sibirica* compose the understory of Qilian juniper forest (Wang et al., 2012). As two dominant conifer species in arid northwestern China, Chinese pine, and Qilian juniper grow in harsh conditions and are particularly sensitive to climate change. These two species play a crucial role in reconstructing regional climate variations (Kang et al., 2012; Yang et al., 2014).

The region has a typical continental climate with relatively dry and cold winters but wet and warm summers. In HSM, the long-term climate records (1951–2012) from the Jingyuan station, the nearest national meteorological station (36°34′ N, 104°41′ E, 1,398 m a.s.l.), show a mean annual temperature of 9.1°C, with an annual precipitation of 231.1 mm. January and July are the coldest and warmest months, with temperatures of −7.2 and 22.7°C, respectively. More than 80% of the annual precipitation is received during May-September. Long-term climate records (1957–2012) from the national meteorological station Qilian (38°11′ N, 100°15′ E, 2,787 m a.s.l.), which is located at a distance of 40 km from SDL, show a mean annual temperature of 1.1°C and an annual precipitation of 404.8 mm. Mean January and July temperatures are −13.1 and 13.1°C, respectively. May-September rainfall accounts for 90% of the annual precipitation (Zeng and Yang, 2016).

### Tree selection and sampling

Nine Chinese pines in HSM and eight Qilian junipers in SDL were randomly selected. All trees were dominant individuals with healthy and upright stems. Three groups were successively defined, based on tree characteristics (age and size) to disentangle the effect of size and age on xylem phenology. Two microcores (15 mm in length, 2 mm in diameter) per tree were extracted weekly from April to October during 2013 and 2014 using a Trephor (Rossi et al., 2006a). Sampling starting at breast height (1.3 m) on the stem and followed a spiral pattern. The distance between each sample position was at least 2–5 cm horizontally and 2–3 cm vertically to avoid the resin ducts induced during the previous samplings (Forster et al., 2000). Before sampling, the dead outer bark was removed and samples generally involved the previous three to five tree rings and the current growth layers with the cambial zone and adjacent phloem (Rossi et al., 2006b). The microcores were placed in Eppendorf microtubes with ethanol 50% in water, and stored at 5°C to avoid tissue deterioration.

### Sample preparation and data collection

The microcores were embedded in paraffin and cut in transverse sections using a rotary microtome. The sections (10–12 μm in thickness) were stained with a 1% water solution of safranin and astra blue (Oladi et al., 2011) and observed using a light microscope under bright field and polarized light at 200–400× magnifications.

We measured the number of (1) cambial, (2) radial enlarging, (3) secondary wall thickening, and (4) lignifying and mature cells along three radial files (Figure 1). Cambium showed small radial diameters and thin primary cell walls (Rossi et al., 2006b). Enlarging cells contained protoplast and had thin primary cell walls, but with a radial diameter greater by a factor of two compared to cambial cells. During this phase, we found that cells were often deformed and fragile during manipulation. The onset of secondary wall thickening was identified under polarized light. The arrangements of cellulose microfibrils between enlarging and secondary wall thickening tracheids were different when observed under polarized light: cells composed of primary walls were dark, whereas cells with secondary walls shined (Rossi et al., 2008). The lignification started from cell corners and was characterized by the color changing from blue to red. The cells were considered mature when the protoplast was lost, tracheid lumen was empty, and the cell walls turned red.

The onset and ending of each phenological phase of xylem was assessed and calculated in days of the year (DOY). In spring, when at least one cell was observed in enlargement, xylem formation was considered to have started. In late summer, when the last formed cell was completely mature, wood formation was considered complete (Rossi et al., 2014). We calculated the duration of wood formation as the number of days between the beginning of the enlarging phase and the end of lignification.

### Statistics

The overall differences in the timings of xylem phenology (i.e., the beginning and end dates of each differentiation phase) were analyzed using multivariate analysis of variance (MANOVA). *Post-hoc* multiple comparisons between groups were performed with orthogonal contrasts. Moreover, cell production and the duration of the differentiation phases were compared between groups using repeated measurements mixed models with trees included as a random factor. Given that the number of sample trees differed between groups, we conducted multiple comparisons with least-squares means adjusted for unbalanced data based on the approximation described by Kramer (1956). All statistics were performed using SAS 9.4 (SAS Institute Inc., Cary, NC).

## Results

### Tree characteristics

**Figure 2:**
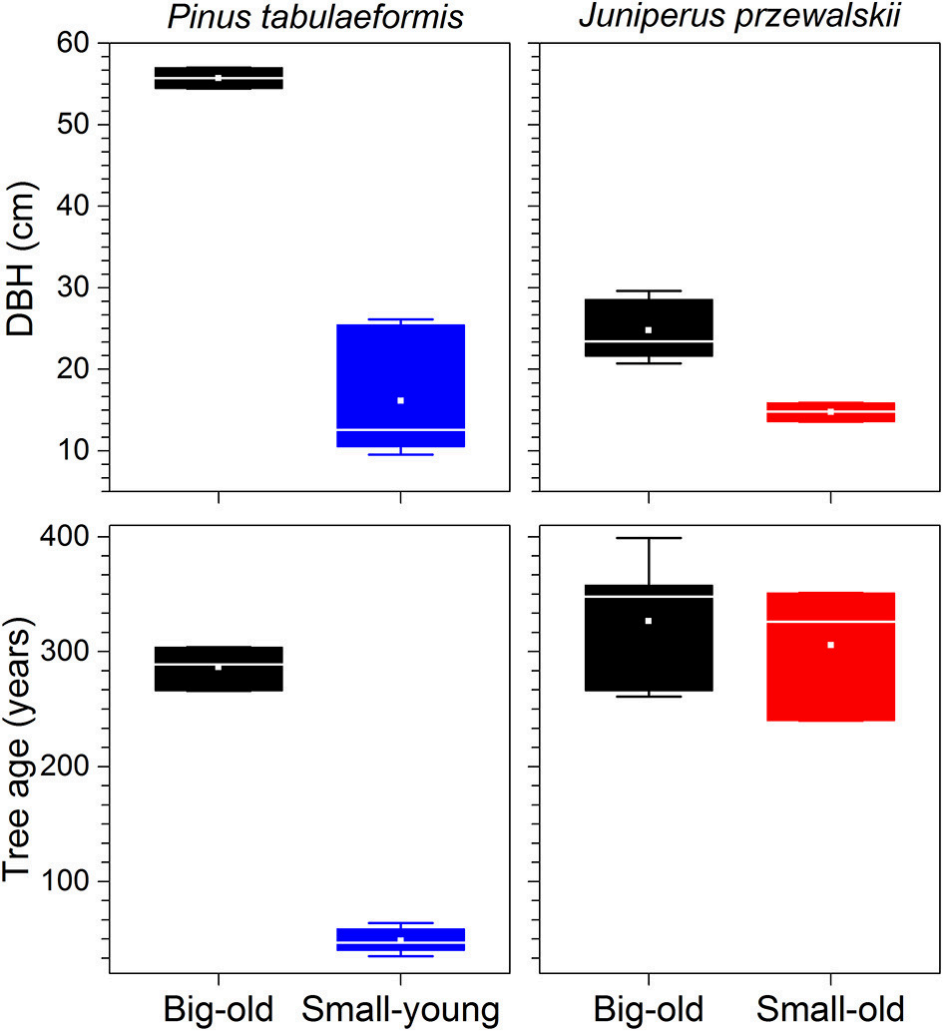
Tree characteristics of each species investigated. Boxes represent upper and lower quartiles; whiskers achieve the 10th and 90th percentiles; the median is drawn as a horizontal white line; and the average value is shown as a white square.

Out of the nine Chinese pines selected for this study, six were small-young trees with an average age of 49 ± 10 (mean ± standard deviation) yr and a diameter at breast height (DBH) of 16 ± 7 cm (Figure 2). The three remaining Chinese pines were big-old trees with an age of 286 ± 16 yr and 56 ± 1 cm DBH. The Qilian junipers were of different sizes but of similar age (319 ± 53 yr). Five out of the eight were defined as big-old trees with 25 ± 4 cm DBH. The three remaining were defined as small-old trees with 15 ± 1 cm DBH (Figure 2).

### Cambial activity

**Figure 3:**
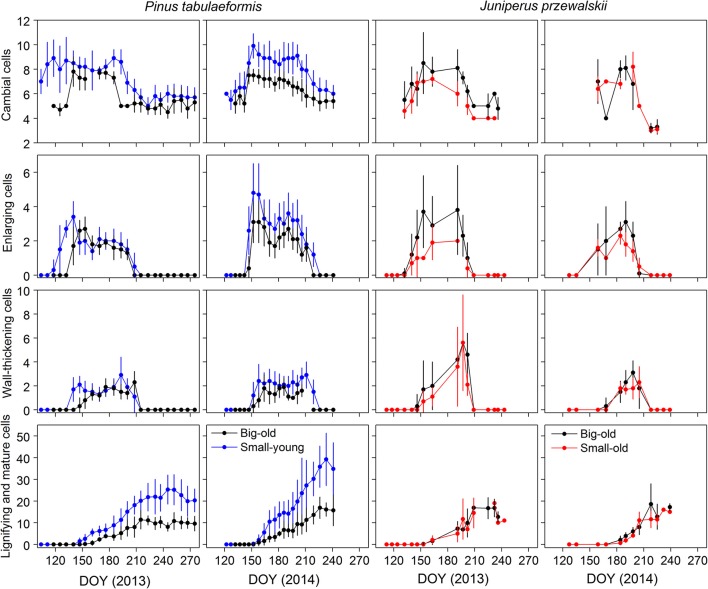
Number of cells during the different phases of xylem differentiation of trees for the different groups and sites in 2013 and 2014. Error bars indicate standard deviations for sampling trees per sampling date.

The same variation in cell numbers was observed between tree size and age, although the dynamics were seldom delayed (Figure 3). During winter, the dormant cambium contained 4–6 cells. The number of cambial cells increased from 7 to 10, which indicated the beginning of cell division. In 2013, we observed cambial activity on 21 April (DOY 111) in small-young pines, almost 1 month earlier than in big-old pines. The cambial cells returned to the quiescent phase in late July and early August (DOY 207–214) in big-old and small-young pines, respectively. In 2014, cambial activity occurred in late spring (DOY 142–147) and ceased in early August (DOY 218–224) in both groups of pines. Cambial cells of big-old and small-old junipers started to increase in early May (DOY 126–133) in 2013, which was nearly 4 weeks earlier compared to 2014. For this species, the termination of cambial activity was observed in late July and early August (DOY 201–216) for both study years.

### Cell differentiation

The general pattern of xylem formation was similar among trees and years, although we observed different timings for the differentiation phases between the groups (Figure 3). During the two growing seasons, the number of cells in enlarging and wall-thickening phases formed bell-like curves, while mature cells increased until a plateau was reached. For pines, the number of cells during the 2013 enlargement phase increased earlier in small-young trees compared to big-old trees. This pattern also occurred during wall thickening and lignification. However, in 2014, both groups started each phenological phase at the same time. During the two growing seasons, small-young pines produced more cells than big-old pines. Both groups (small-old and big-old) of junipers exhibited similar variation in the number of xylem cells for the 2 years.

### Timings of xylem phenology

**Figure 4:**
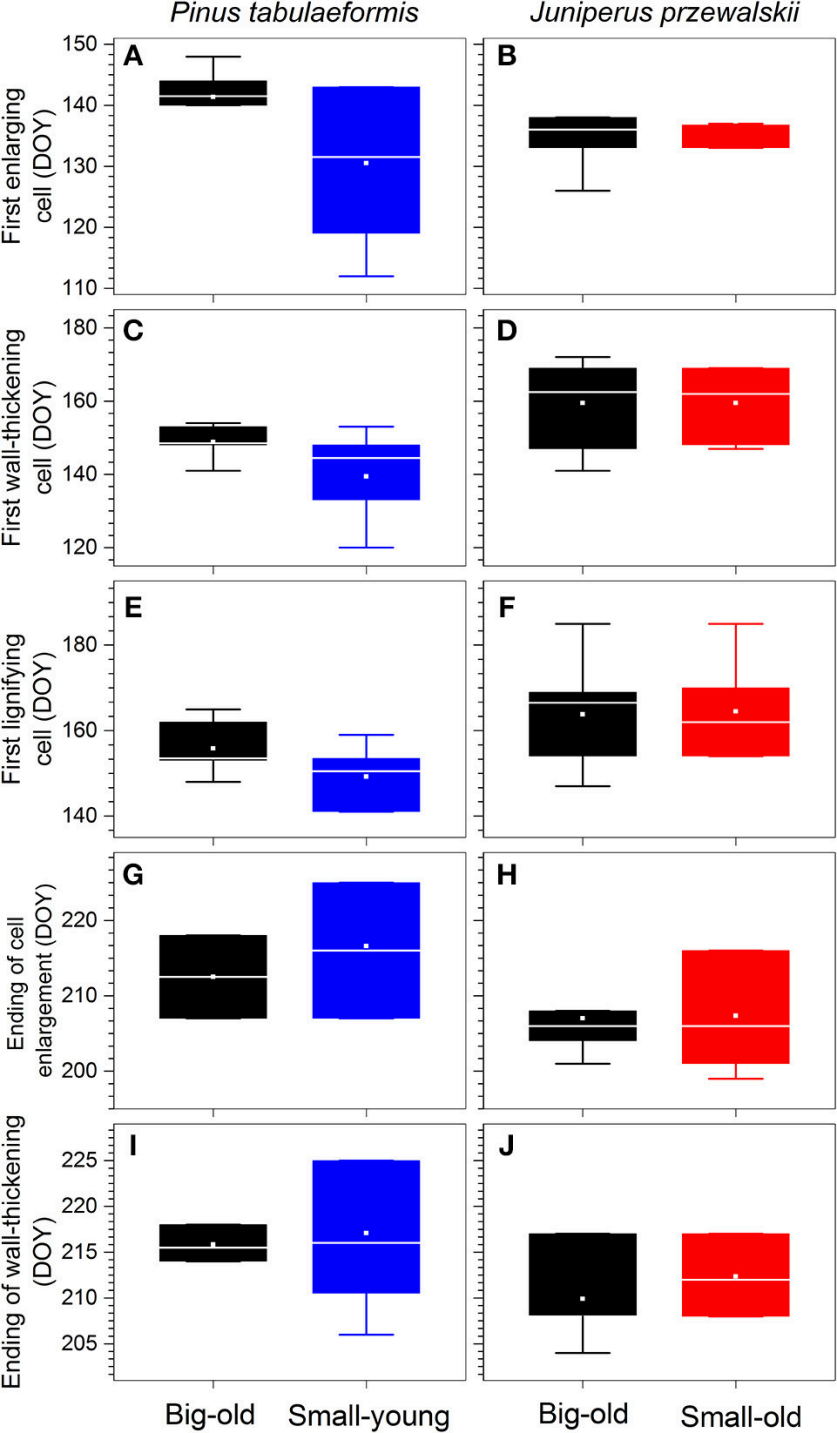
Timings of xylem differentiation of each species. Boxes represent upper and lower quartiles; whiskers achieve the 10th and 90th percentiles; the median is drawn as a horizontal white line; and the average value is shown as a white square.

For pines, the first enlarging cells were observed in early May (DOY 131) in small-young trees with a larger variation within group, almost 10 d earlier compared to big-old trees (Figure 4A). We also detected the same trend in the timings of cells undergoing wall thickening and lignification, but with gradually decreasing differences (Figures 4C,E). The termination of cell enlargement occurred in early August (DOY 213–217; Figure 4G). For junipers, both groups started xylem differentiation in mid-May (DOY 137; Figure 4B) and terminated in late July (DOY 207; Figure 4H). The first wall-thickening and lignifying cells were observed in early June (DOY 160–165; Figures 4D,F). Despite the variability within groups, most wall thickening was complete by late July and early August (DOY 210–217) for all trees and sites (Figures 4I,J).

MANOVA detected a significant difference in xylem phenology among the four groups of trees (Wilk's lambda = 0.15, *p* < 0.0001). Pines were significantly different from junipers (*p* < 0.05 or lower, Table 1) except for the big-old pines vs. the small-old junipers (Wilk's lambda = 0.66, *p* > 0.05). For pines, we found that big-old trees were different from small-young trees (Wilk's lambda = 0.59, *p* < 0.05). Differences were not significant when small-old junipers were compared with big-old junipers (Wilk's lambda = 0.91, *p* > 0.05; Table 1).

**Table 1:** *Post-hoc* multiple comparisons of xylem phenology between groups conducted by orthogonal contrasts.

	**Small-young pine**	**Big-old juniper**	**Small-old juniper**
Big-old pine	0.59^*^	0.53^**^	0.66
Small-young pine	–	0.22^***^	0.32^***^
Big-old juniper		–	0.91

*Pairwise values correspond to Wilk's lambda in multivariate analysis of variance (MANOVA). One, two, and three asterisks correspond to p < 0.05, p < 0.01, and p < 0.001, respectively*.

### Cell production and duration of cell differentiation

All models that compared the duration of xylem formation and cell production were significant with *F*-values ranging between 5.85 and 29.09 (*p* < 0.01 or lower, Table 2). Overall, the young trees differed from the old trees, while few differences were observed between big-old trees and small-old trees. Cell enlargement, which corresponds approximately to the period of cell division in cambium, lasted between 68.1 and 86.0 d. The young trees exhibited a significantly longer cell enlargement period compared to the old trees. Pines required a longer period for wall thickening to complete compared to junipers, with small-young pines undergoing a longer wall thickening phase (77.6 d) compared to big-old pines (67.0 d). On average, 76.7 d were required to complete the differentiation of xylem, but the young trees exhibited significantly longer periods of xylem differentiation. Young trees produced 33.9 cells, which was far greater compared to the old pines and junipers (Table 2).

**Table 2:** Comparisons between the duration of the differentiation phases and the xylem cell production in trees of *Pinus tabulaeformis* and *Juniperus przewalskii*.

	**Model**		***Pinus tabulaeformis***		***Juniperus przewalskii***	
	**F**	**P**	**Big-old**	**Small-young**	**Big-old**	**Small-old**
Cell enlargement (days)	8.66	<0.001	71.1 ± 3.6^a^	86.0 ± 6.4^b^	68.1 ± 9.9^a^	70.0 ± 9.6^a^
Wall thickening (days)	29.09	<0.0001	67.0 ± 3.4^a^	77.6 ± 6.6^b^	50.4 ± 10.1^c^	52.8 ± 6.3^c^
Xylem differentiation (days)	5.85	<0.01	74.5 ± 3.6^a^	86.5 ± 6.8^b^	71.0 ± 10.5^a^	75.0 ± 7.2^ab^
Xylem production (cells)	9.40	<0.001	14.4 ± 4.5^a^	33.9 ± 13.9^b^	16.9 ± 7.0^a^	13.7 ± 4.3^a^

*Values followed by the same letter are not statistically different (p > 0.05)*.

## Discussion

This study compared the intra-annual dynamics of cambial activity and xylem formation in two conifer species with different ages and sizes growing in the semi-arid region of northwestern China. We specifically designed our monitoring approach to separate the effects of these two factors, which are commonly entangled within trees. We found that small-young pines exhibited different timings of xylem formation. In particular, we observed earlier cambial reactivation (2013) and later cell differentiation (2014) compared to big-old pines. However, these differences disappeared when comparing xylem phenology between junipers of different sizes but similar age. Although the initial hypothesis that xylem phenology is size-dependent could not be completely rejected, our results suggest that the effect of age can play an important role in the timing and duration of xylem formation.

### Influence of age on xylem phenology

Endogenous factors play an important role in growth resumption (Perrin et al., 2017). Begum et al. (2010) found that under localized heating, the cambial reactivation in *Cryptomeria japonica* within a temperate ecosystem occurred earlier in 55-year-old trees compared to 80-year-old trees. They pointed out that cambial age may affect cambial sensitivity to air temperature in spring, which could explain the age-related timings of cell differentiation in our study. Furthermore, we observed an earlier onset of cambial reactivation in small-young pines in 2013, which may indicate a higher capacity for growth under lower temperatures than big-old pines. On the other hand, as observed by Arco Molina et al. (2016), bark thickness increases with cambial age, resulting in an insulating and protective ability which deceasing the possibility of old trees suffering from frost damage. Consequently, the cambial reactivation in old trees may also be delayed, corresponding to the gradual warming that occurs in spring, owing to the potential effect of bark isolation (Rossi et al., 2008).

The variation in xylem phenology between young and old trees may be due to different survival strategies. Younger trees converge their resources to enhance their survival during establishment, as demonstrated by the higher rates of photosynthesis and transpiration relative to older trees, especially under severe environmental conditions (Bond, 2000). The earlier reactivation of bud growth in younger trees increases the capture of sunlight and gains in carbon before being shaded by dominant individuals, strengthening the viability and competitiveness to survive in the understory, although this increases the potential risk that new growth tissues will be damaged by late spring frost (Lopez et al., 2008; Vitasse, 2013). On the contrary, older trees appear to have a more conservative strategy, with later growth reactivation (e.g., leaf unfolding; Vitasse, 2013), which protects them from frost damage in the spring and guarantees a safe environment for developing vegetative and reproductive meristems.

It has been suggested that the auxin produced from young developing needles or buds that are basipetally transported into stems influences cambium reactivation (Aloni, 2001). Based on this hypothesis, it could be deduced that the earlier start of wood formation in younger trees may be the result of the shorter distance between source (leaves) and sink (stem; Rossi et al., 2008). However, Li et al. (2016) analyzed young (20–40 years old) and mature (70–110 years old) trees, discarding the effect of tree height on the onset of cambium activity in spring. Thus, the hypothesis of a longer distance from the crown is not able to explain the resumption of later growth in older trees. Cambial resumption can occur before or without leafing (Begum et al., 2007; Rossi et al., 2009), demonstrating that the auxin produced in developing buds is not compulsory for cambium reactivation (Sundberg et al., 1991).

### Influence of age on tree growth

It has been suggested that an early onset of cambial reactivation increases the time available for cell differentiation, resulting in higher cell production along a radial file (Rossi et al., 2008; Lupi et al., 2010; Rathgeber et al., 2011). In this study, cambial activity and cell differentiation of young pines started earlier in 2013, with the duration of wood formation lasting ~3 weeks longer relative to the old pines. In 2014, cell differentiation lasted 1 week longer in young pines. Accordingly, these younger pines exhibited higher cell production compared to the older pines. This finding confirmed the observations by Rossi et al. (2008) and Li et al. (2013), in which an earlier onset of xylogenesis in young trees corresponds to higher cell production and longer durations of cell differentiation. Compared to young trees, older trees allocate resources mainly for fitness and defense rather than for growth (Herms and Mattson, 1992; Johnson and Abrams, 2009), which can also explain the lower cell production of older trees regardless of their size. We observed that cell production in younger trees doubled (by 15 d) with a lengthening of the growing season, corresponding to 17% of the growing season of older trees, which confirmed the non-linear relationship between cell production and the duration of xylogenesis (Rossi et al., 2013). Under harsh environmental conditions, the marginal lengthening of the cell division period can also correspond to higher rates of growth and lead to disproportionate increases in xylem cell production (Rossi et al., 2014). Moreover, young trees have wider tree-ring widths compared to old trees (Shi et al., 2015), which may also explain the need for a longer growing season.

In our study, the old trees were older than >280 years, exceeding the ages of trees reported in other studies by a significant margin (<120 years; De Luis et al., 2009; Rathgeber et al., 2011; Vieira et al., 2013). As trees age, physiological processes, such as foliar efficiency, photosynthetic rates, and gas exchanges, decrease accordingly (Day et al., 2001; Mencuccini et al., 2005). Ryan and Waring (1992) suggested that a lower photosynthetic rate would account for the decline in aboveground wood production in older trees. Thus, the differences in results between our study and other studies could be related to the younger ages of the trees studied in these previous investigations.

Our study included trees belonging to two different species, which may have influenced the results of this investigation; namely, the differences in size between the two classes of pines were greater than those compared to junipers. Therefore, the changes in xylem phenology in junipers could be potentially influenced by the amplitude of the differences in tree sizes. Nevertheless, our experimental design was a first attempt to disentangle the effects of age and size on xylem phenology on a short timescale. Our results indicated that tree age should be carefully considered in growth-climate relationships. For example, tree-age classifications should be included in plant physiological modeling (Shishov et al., 2016; Yang et al., 2017) to reliably simulate growth processes under specific climatic drivers.

## Conclusion

The climatic signals that directly affect trees can be registered by xylem during its growth. If the timings and duration of xylem formation change, xylogenesis could occur under different environmental conditions and thus be subject to different climatic signals. Specifically, small-young pines exhibited different timings of xylem formation, in particular, earlier cambial reactivation in 2013 and later cell differentiation in 2014, compared to big-old pines. However, these differences disappeared altogether when comparing xylem phenology between junipers of different sizes but similar age. Different growth timings could modify the growth-climate relationships along the lifespan of trees. For example, spring frost could affect earlier young tree growth. On the other hand, with the later onset of wood formation, older trees are more likely to avoid frost events and subsequently frost damage. Even if the effect of size cannot be fully eliminated by our experimental design in the field, the study demonstrated that tree age does play an important role in timings and duration of growth. Thus, the age of trees should be taken into account when investigating the response of trees to their environment to provide unbiased estimations of climate growth relationships.

## Author contributions

QZ, SR, and BY: conceived the ideas that led to this investigation; QZ: conducted the experiment; SR: designed the methodology; QZ and SR: analyzed the data. All authors contributed to the writing of this manuscript.

### Conflict of interest statement

The authors declare that the research was conducted in the absence of any commercial or financial relationships that could be construed as a potential conflict of interest.
